# Predicting the Local Response of Metastatic Brain Tumor to Gamma Knife Radiosurgery by Radiomics With a Machine Learning Method

**DOI:** 10.3389/fonc.2020.569461

**Published:** 2021-01-11

**Authors:** Daisuke Kawahara, Xueyan Tang, Chung K. Lee, Yasushi Nagata, Yoichi Watanabe

**Affiliations:** ^1^ Department of Radiation Oncology, Institute of Biomedical & Health Sciences, Hiroshima University, Hiroshima, Japan; ^2^ Department of Radiation Oncology, University of Minnesota-Twin Cities, Minneapolis, MN, United States

**Keywords:** radiomics, machine learning, brain metastases, gamma knife, radiosurgery, local control

## Abstract

**Purpose:**

The current study proposed a model to predict the response of brain metastases (BMs) treated by Gamma knife radiosurgery (GKRS) using a machine learning (ML) method with radiomics features. The model can be used as a decision tool by clinicians for the most desirable treatment outcome.

**Methods and Material:**

Using MR image data taken by a FLASH (3D fast, low-angle shot) scanning protocol with gadolinium (Gd) contrast-enhanced T1-weighting, the local response (LR) of 157 metastatic brain tumors was categorized into two groups (Group I: responder and Group II: non-responder). We performed a radiomics analysis of those tumors, resulting in more than 700 features. To build a machine learning model, first, we used the least absolute shrinkage and selection operator (LASSO) regression to reduce the number of radiomics features to the minimum number of features useful for the prediction. Then, a prediction model was constructed by using a neural network (NN) classifier with 10 hidden layers and rectified linear unit activation. The training model was evaluated with five-fold cross-validation. For the final evaluation, the NN model was applied to a set of data not used for model creation. The accuracy and sensitivity and the area under the receiver operating characteristic curve (AUC) of the prediction model of LR were analyzed. The performance of the ML model was compared with a visual evaluation method, for which the LR of tumors was predicted by examining the image enhancement pattern of the tumor on MR images.

**Results:**

By the LASSO analysis of the training data, we found seven radiomics features useful for the classification. The accuracy and sensitivity of the visual evaluation method were 44 and 54%. On the other hand, the accuracy and sensitivity of the proposed NN model were 78 and 87%, and the AUC was 0.87.

**Conclusions:**

The proposed NN model using the radiomics features can help physicians to gain a more realistic expectation of the treatment outcome than the traditional method.

## Introduction

Approximately 5 to 40% of cancer patients are diagnosed with a metastatic brain tumor during their treatment. Furthermore, patients have brain metastases (BMs) ten times more often than primary malignant tumors of the brain (1, 2). Consequently, BM is the most common brain tumor treated by radiation therapy. Whole-brain radiation therapy (WBRT) and stereotactic radiosurgery (SRS) are regularly offered to manage BMs. The techniques are effective with improved local control of tumors and more prolonged survival of patients (3). The RTOG-9508 study compared the treatment responses of WBRT alone, SRS alone, and WBRT plus SRS for the BMs (4). For the WBRT alone or WBRT plus SRS, the total prescribed dose of WBRT was 37.5 Gy with 2 to 5 Gy per fraction. For the SRS treatment, the prescribed dose was assigned from an earlier dose-escalation RTOG radiosurgery trial (90–05) (5). The mean survival time did not differ much among the three techniques. The local control rate at three months after WBRT plus SRS or WBRT alone ranged from 71 to 82%, indicating about 20 to 30% failure rate. Hence, a predictive capability of the radiation therapy outcome of BMs may provide a decision tool to clinicians for the effective management of patient care with the most desirable treatment outcome. If the local failure is predicted for radiotherapy, the treatment plan can be modified to improve the local control by, for example, increasing the dose.

There are several prognostic tools or prognostic indices, specially developed for the radiation therapy of BMs such as the RTOG Recursive Partitioning Analysis (RPA), the Score Index for Radiosurgery (SIR), the Basic Score for Brain Metastases (BSBM), and the Graded Prognostic Assessment (GPA) (6). These indices are proven to have clinical value for predicting the treatment outcome. The addition of more detailed clinical information to the pretreatment characteristics used by the existing prognostic indices might improve the predictive performance. Such new information includes the biological data (*i.e.*, biological markers and genomics) specific to the patient (7) and the quantitative imaging data obtained by radiomics (8–10).

Radiomics analyzes the medical image quantitatively to explore features unique to a patient (11). It has been used for classifying patients and evaluating their risk to customize oncological treatments (12, 13). Some researchers used radiomics to find the correlation between radiomics signatures and radiation treatment outcome (14–16). Zhou et al. tried to predict survival after chemotherapy of glioblastoma patients using several imaging features based on MR image (17). Ryu et al. performed a prognostic prediction using features obtained from functional images (18). Other studies have combined radiomics with genomics to associate radiomics features with gene mutations that are clinically proven to predict therapy response (19). A recent study reported that radiomics features could potentially be used as surrogate biomarkers for predicting tumor prognosis following Gamma Knife radiosurgery (GKRS) (20).

Goodman et al. categorized brain tumor images into three groups: homogeneous, heterogeneous, or ring-enhancing (21). They found that these enhancement patterns are significant prognostic factors in the response of brain metastases after radiosurgery. A drawback of their approach is the subjective nature of the classification technique. Visual classification into one of the three patterns is often neither possible nor accurate because real images do not display clear ring-like features or completely uniform pixel colors throughout the tumor.

In the current study, therefore, we proposed the application of radiomics and a machine learning (ML) technique to create a more reliable and accurate method than the decision with the visual evaluation for predicting the treatment outcome, in particular, the local response of the tumor to radiation therapy. Primarily, we built a model to predict the response of metastatic tumors treated with GKRS.

## Materials and Methods

### Patients

Previously, we analyzed the treatment outcome of 88 patients with either renal cell or melanoma cancer as the primary disease, who underwent GKRS at the University of Minnesota from 2005 to 2012 for their BMs (22). For the current study, we selected a subset of the patients, 45 melanoma patients with a total of 115 tumors, for model building. Furthermore, we obtained the new data of nine melanoma patients with a total of 42 tumors from the database of GKRS patients treated from 2013 to 2017 for the final evaluation of the model. The characteristics of the patients and their tumors are presented in **Table 1**.

**Table 1:** Patient characteristics.

		Model building dataset	Model evaluation dataset
Number of patients		45	9
Number of tumors		115	42
Age (years)	Median	61.5	59.5
	[Range]	[32–86]	[42–71]
Gender	Male	23 (53%)	5 (56%)
	Female	20 (47%)	4 (44%)
Local Response (LR)	Group I: CR + PR Group II: SD + PD	83 32	35 7
Tumor volume (cc)	<4.2	102 (89%)	40 (95%)
	≥4.2 to ≤14.1	12 (10%)	2 (5%)
	>14.1	1 (1%)	0 (0%)

### Image Acquisition

All patients were scanned with a 1.5T MRI (Siemens Syngo MR) scanner. The total scanning time was about 15 min for the whole brain scan. We used the Siemens 12 channel head matrix coils. The scanning protocol was a FLASH (3D fast, low-angle shot) with gadolinium (Gd)-contrast enhanced T1-weighting. The scan parameters are shown in **Table 2**.

**Table 2:** MRI scan parameters of the Fast Low Angle Shot (FLASH) pulse sequence.

Parameter	Values
TE	4.76 ms
TR	9.4 ms
Echo train length	1
Number of acquisitions	1
Bandwidth	260 kHz
Flip angle	25°
FOV	256 mm × 256 mm
Voxel size	0.5 mm × 0.5 mm × 1.0 mm
Slice thickness	1 mm

### Treatment

We treated patients with the Leksell Gamma Knife Model 4C (Elekta AB, Stockholm, Sweden). The prescription dose was decided based on tumor size according to the RTOG 90-05 trial protocol (5). The prescription isodose level varied from 40 to 80%, with a medium of 50%. The prescribed dose to the gross tumor volume (GTV) was 24 Gy for the tumor volume <4.2 cc, 18 Gy for the tumor volume ≥4.2 cc to ≤14.1 cc, 15 Gy for the tumor volume >14.1 cc. **Table 1** shows the number of tumors in these three-volume ranges.

### Follow-Up

Patients after GKRS were followed at 3-month intervals with MRI performed at each visit. The time from the first SRS to the last follow-up imaging study or death was defined as patient follow-up duration.

### Treatment Response Evaluation

To evaluate the local response (LR) of the tumors to the treatment, we measured the maximum lengths of a tumor in three orthogonal directions using pretreatment and follow-up MRI images. Tumor volumes were calculated with the ellipsoid volume formula. The LR status of treatment was determined by using the latest available follow-up imaging study at the time of the data collection. The medium follow-up length was 7.6 months. The status of each tumor was evaluated based on modified RECIST criteria (23). A tumor was defined as progressive disease (PD) if there was a relative increase in tumor volume on follow-up MRI by greater than 20% compared to pretreatment MRI. Lesions in which volume increased less than 20% or decreased less than 30% of pretreatment were considered a stable disease (SD). The tumor, whose size fell more than 30%, but it was still visible on the follow-up MRI, was categorized as a partial response (PR). Any lesion which disappeared on the MRI was considered as complete repose (CR). We accepted only conservative management of cancer during the follow-up period to be included in the analysis. To enhance the predictive performance, we classified the LR into two groups as follows: response group (CR + PR) and non-response group (SD + PD). The LR data of the patients are presented separately for model building and model evaluation datasets in **Table 1**.

For the patients in the current study, we did not do either additional imaging study to delineate necrotic areas or took tissue samples for a histopathological examination. Instead, to minimize the volume measurement error due to the necrosis, we examined the available T1-weighted Gd-contrast enhanced MRI to identify necrosis by the existence of the edema around the enhanced lesion or clear hemorrhage inside the lesion, or by checking the patient’s neurologic symptom. We did not see these indications among the patients and their tumors, which we used for the current study. Thus, our tumor volumes might contain necrosis or hemorrhage inside the volume unless it was present clearly outside of the tumor.

### Radiomics Analysis

**Figure 1:**
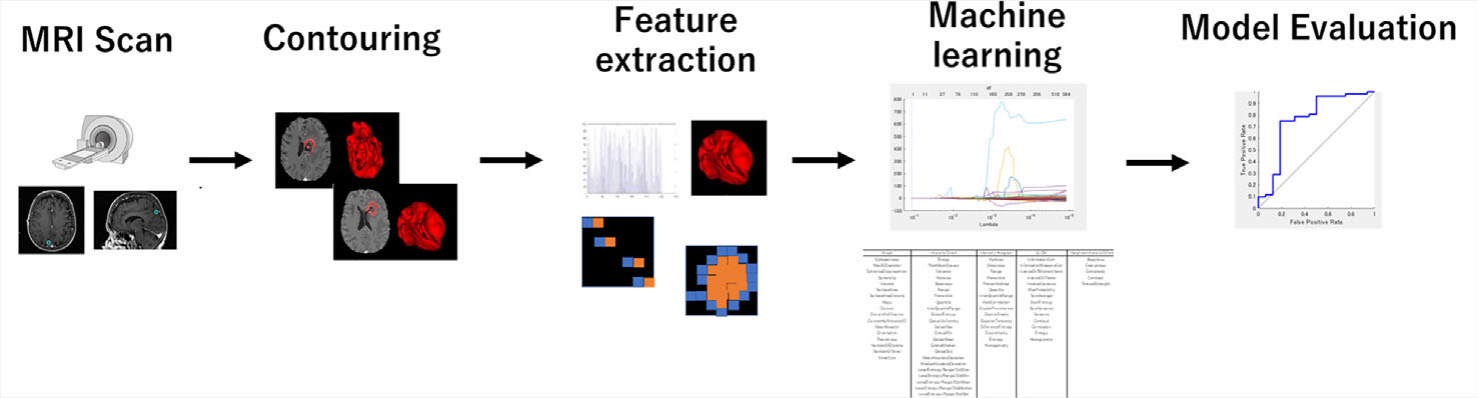
The process for generating a prediction model using a machine learning method with the radiomics feature. The radiomics feature was extracted from the treatment planning MRI data with IBEX. LASSO analysis provided a more regularized model by reducing the number of features. The machine learning model used neural networks.

The process of the radiomics analysis is shown in **Figure 1**. The pixel values of the MRI data were rescaled by using the RescaleSlope and RescaleIntercept tags from the DICOM header as follows:

(1) Image Data=(Image Data) × RescaleSlope + RescaleIntercept+1000

Before calculating radiomics features, we applied the medium smooth filter to the rescaled image data. All treatment planning MRI images were analyzed to extract textural features from the GTVs contoured for the radiotherapy plans. The GTV was manually contoured for the radiosurgery treatment planning by radiation oncologists. The feature extraction was performed using IBEX software (24). It is noted that the tumors smaller than 4 mm diameter or volume of 33.5 mm^3^ were excluded from further study because of its limited number of pixels available for the texture analysis. We used the following six different radiomics feature classes: Gradient Orient Histogram (GOH) (35 features), Gray-Level Co-occurrence Matrix (GLCM) (594), Gray-Level Run Length Matrix (GLRLM) (33), Intensity Direct (ID) (55), Neighbor Intensity Difference Matrix (NIDM) (5), and Shape (18). The resulting 740 features were considered in this study. When there was an option of 2.5D or 3D analysis for texture calculations, we selected 2.5D.

The least absolute shrinkage and selection operator (LASSO) regression was performed in the MATLAB program (Mathworks, Natick, MA, USA) to select the suitable features for the prediction. The LASSO regression performs feature selection during model construction by penalizing the respective regression coefficients. As this penalty is increased, more regression coefficients shrink to zero, resulting in a more regularized model. The most significant predictive features were selected from among all the candidate features for the subsequent training session to build an ML-based prediction model.

### Machine Learning-Based Prediction Model

**Figure 2:**
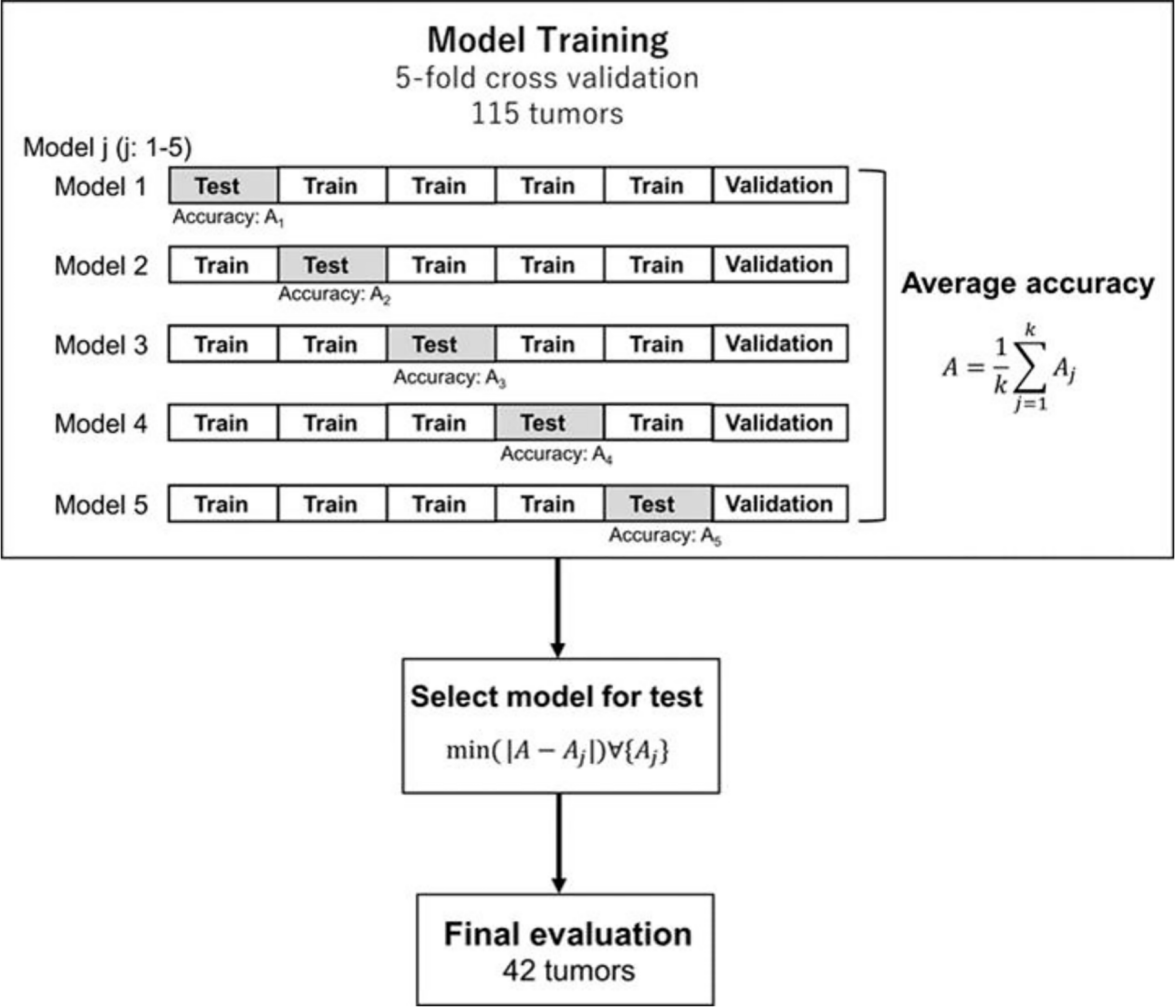
Generation and testing of the prediction model. The proposed NN model with five-fold cross-validation was built in the model training section. Then, the model, which was the closest to the average accuracy of the five prediction models, was selected. The selected model was used for the final evaluation with 42 tumors in the model evaluation dataset.

**Figure 2** shows an overview of the prediction model generation. A machine learning (ML)-based model was built by using a neural network (NN) with ten hidden layers and rectified linear unit activation (ReLU), as implemented in the MATLAB program. For the classification of local response, tumors in the response group (PR + CR) were labeled as 1, and tumors in the non-response group (SD + PD) were labeled as 0. For the model training, we used the data in the model building dataset (115 tumors of 43 patients) shown in **Table 1**. Tumors were randomly partitioned into a training set (55% tumors), a validation set (15% tumors), and a testing set (30% tumors). The predictive model for the classification was created with the training set and the validation set. The performance of the predictive model was evaluated by the testing set by calculating the accuracy and sensitivity of the prediction. The training-validation-testing processes were repeated five times for the five-fold cross-validation. Then, a model that was the closest to the average accuracy of five-fold cross-validation was selected for the final evaluation. We performed the final assessment with the data in the model evaluation dataset (42 tumors of nine patients), as shown in **Table 1**. The predictive performance of the models was assessed using the area under the receiver operator characteristic (ROC) curve, AUC, as well as the accuracy and sensitivity.

### Visual Evaluation

Goodman et al. classified the lesion characteristics into homogeneous, heterogeneous, or ring-enhancing by the pattern of enhancement (20). The uniform enhancement of the entire lesion was defined as homogeneous. If there were any areas of nonhomogeneous enhancement, it was defined as the heterogeneous. Additionally, if there was a rim or ring of contrast enhancement surrounding a central non-enhancing low-signal intensity area, it was identified as a ring-enhancing. In the current study, an experienced radiation oncologist classified the tumors into three types of patterns (homogeneous, heterogeneous, or ring-enhancing) by visually inspecting the MR images. The treatment outcome was predicted based on the image. Nieder et al. showed that important prognostic factors for complete remission were the small volume and no necrosis (25). Based on the well-accepted knowledge (20, 25), we assigned the predicted response of the homogeneous tumors to the response group (group I) and tumors with heterogeneous or ring enhancement to the non-response group (group II). We compared the visual evaluation method and the ML method using the data in the model building dataset (115 tumors of 42 patients).

## Results

**Figure 3:**
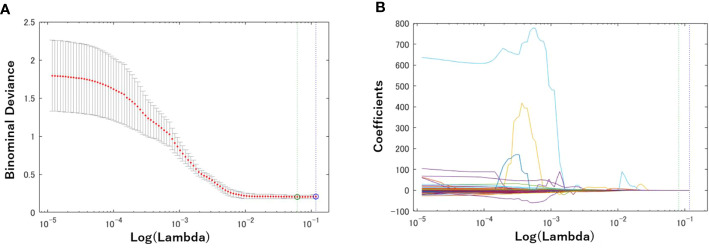
Radiomics feature selection using the Lasso logistic regression model. **(A)** Tuning penalization parameter lambda (*λ*) and minimum criterion in the Lasso model. The binomial deviance was plotted versus log (*λ*). **(B)** Lasso coefficient profiles of the 740 radiomics features. The green line showed the optimal lambda in the LASSO method with the least partial likelihood deviance.

First, a total of 740 radiomics features were extracted from the BM MRI images. Then, the number was reduced to seven features by using the LASSO regression method. **Figure 3** shows the binomial deviation (a) and the coefficients (b) as a function of the tuning penalization parameter *λ* for the LASSO linear regression. As *λ* increased, only a few coefficients of 740 features remained non-zero, indicating only parameters important for an accurate model. The selected features were 45-7ClusterShade, 225-7ClusterShade, 45-7InformationMeasureCorr-1, 225-7InformationMeasureCorr-1, 90-4InformationMeasureCorr-2, 225-7Energy, and 315-5Energy.

**Figure 4:**
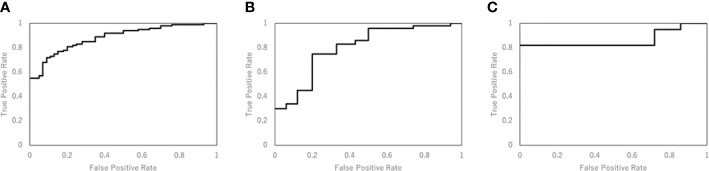
The performance of the NN model was validated according to the ROC metrics for **(A)** the training data in model training section, **(B)** the testing data in model training section, and **(C)** for the final evaluation dataset. The AUC score was 0.89 for the training data, 0.82 for the testing data, and 0.87 for the final evaluation dataset.

**Table 3** shows the performance of the NN models. There were five models generated in the five-fold cross-validation step. Those models were evaluated with the training and testing datasets separately. The average accuracy of the five models was 0.80, with the training data. The model closest to the average accuracy was model 3. Hence, the final evaluation was performed with the model 3. The accuracy and sensitivity of the final model were 0.78 and 0.87 with the model evaluation dataset. **Figure 4** shows the performance of the classifier according to the ROC metrics for the training and testing datasets. The AUC score was 0.89 for the training data and 0.82 for the testing data in the model training section. When we applied the selected model to the final evaluation of 42 tumors in the model evaluation dataset, we obtained the AUC score of 0.87.

**Table 3:** Model performance.

	Training		Test		Final evaluation	
	Accuracy	Sensitivity	Accuracy	Sensitivity	Accuracy	Sensitivity
Model 1	0.74	1.00	0.75	0.76	–	
Model 2	0.79	0.60	0.81	0.75	–	
Model 3	0.80	0.83	0.79	0.78	0.78	0.87
Model 4	0.81	0.60	0.76	0.68	–	
Model 5	0.81	0.70	0.86	0.74	–	
Average	0.80	0.75	0.81	0.75	–	

**Table 4** compares the visual evaluation method and the NN prediction model by accuracy and sensitivity. The former method was applied to the 115 tumors used for the NN model training. The latter was applied to the testing data in the model training section, and the values in the table were the average of the five models. The results showed that the NN model was superior to the visual evaluation for accuracy and sensitivity.

**Table 4:** The assessment of the predictive performance of the visual evaluation and NN model for the testing data used for the NN model training.

	*Visual evaluation*	*NN model*
Accuracy	0.44	0.80
Sensitivity	0.54	0.74

## Discussion

Goodman et al. reported that the pattern of tumor images seen on the Gd-contrast enhanced T1-weighted MR images is valuable for predicting the response of a tumor to radiosurgery (20). The current study used radiomics features extracted from radiotherapy planning MRI (Gd-contrast enhanced T1-weighted) to predict the local response (LR) by a machine learning (ML) method with a neural network (NN) classifier. We compared the predictive performance of the NN model and the visual evaluation method. The accuracy of the new method using the radiomics features yielded a higher prediction accuracy (80%) than the visual approach. Thus, the ML method, such as NN, would be useful for predicting the response of the BMs to GKRS.

The LASSO regression analysis resulted in seven radiomics features, which were useful for the classification, among 740 features initially included in the radiomics analysis. The selection of these features can be understood by the mathematical implication of those features. Cluster shade is a measure of the skewness of the matrix and is believed to gauge the perceptual concepts of uniformity. It may be correlated with lesion characteristics that are heterogeneous or ring-enhancing. Informational Measure of Correlation-1 and Measure of Correlation-1 assess the correlation between the probability distributions using mutual information, which means quantifying the complexity of the texture. Energy is a measure of the magnitude of voxel values in the image. The current study revealed that these were useful features for predicting the response of BMs to GKRS.

The prediction of the LR of BMs to SRS has important practical implications for patients and clinicians. Our prediction model could be useful in clinics. Although the current study created the prediction model of the LR for the radiosurgery, the same approach can be used for all of the treatment methods.

In this study, the comparison between our predictive model and the visual method was made to demonstrate the high predictive performance of the current approach. Goodman et al. (20) tried to identify the necrosis inside the tumor by classifying the tumor into three groups based on the enhancement pattern. However, there is no reliable technique to quantify the amount of necrosis only by visual examination. Hence, the visual classification method suffers from a large uncertainty. Consequently, we expect a large variation among observers for distinguishing three image patterns. Surely, we cannot exclude a potentially better performance of some observers than our method. But, the overall performance of our method should be better than the visual method. Our method does not classify the image pattern into only three types, but it uses more information of images for the decision making than the visual approach. Furthermore, the visual method is applied to only one transverse image since classifying the images into three patterns of the three-dimensional data is time-consuming and almost impossible. As a result, the method should be more accurate for outcome prediction.

There are several recent studies, in which radiomics features were used for more accurate distinction of necrosis from tumor progression and early detection of adverse radiation events (ARE) after radiotherapy of brain tumors (26–29). We used only the GTV (Gd contrast enhanced area) for radiomics analysis in the current study. Suppose we extend the region-of-interest (ROI) by including the volume surrounding the Gd-contrast enhanced area or add other types of imaging data such as PET, for example. In that case, we might be able to predict brain injuries after GKRS. Such a study is interesting and can be undertaken in the future.

There are five limitations to the current study. First, the LR of BMs depends on the prescribed dose. For our GKRS treatment, we prescribed the dose based on the tumor size following the RTOG 90-05 protocol (5). Hence, the LR can be affected not only by the radiomics features but also by the prescribed dose. Secondarily, other clinical factors are statistically significant, but we did not consider in the current study. To improve the prediction performance, therefore, the radiomics features can be combined with the standard biomarkers. Thirdly, the present study used the radiomics features extracted from only radiotherapy planning MRI scans (Gd-contrast enhanced T1-weighted). But, the prediction accuracy may improve by utilizing images taken by other imaging modalities. For example, Wu et al. combined the radiomics features of CT and FDG-PET for predicting distant metastasis in early-stage non-small cell lung cancer after stereotactic body radiation therapy (30). Ordering additional imaging studies other than standards requires additional funding and a special protocol, but it may be an important step for more accurate predictions. Fourth, the current predictive model was built using only the metastatic brain tumors of patients with melanoma as the primary, mainly due to the availability of the treatment follow-up data. Lastly, only one experienced radiation oncologist classified the tumors into three MR image patterns for the visual evaluation. For a fair comparison of the ML-based method with the visual evaluation method, we need to recruit more experts to study the effects of inter-observer variation on the outcome prediction.

To overcome the first three limitations, we plan to improve the prediction model by adding radiomics features of other MR imaging protocols, dosimetric parameters such as prescribed dose and standard biomarkers. Extending the model to BMs with different primary cancer types is straightforward as long as the necessary data for model training are available. The versatile prediction model will be created by including multi-institution and other brain metastases patients. The uncertainty of the inter-observer with visual evaluation is a serious problem. However, we believe that the prediction model proposed in the current study decreases the uncertainty with the visual evaluation.

## Conclusion

The proposed NN model using the radiomics features of tumor image was more accurate than the visual evaluation method using the image pattern information in predicting the local response of brain metastases to GKRS. Because of the excellent prediction ability of the method, the method can be used to help physicians to gain a more accurate prediction of the treatment outcome than the traditional method.

## Data Availability Statement

The raw data supporting the conclusions of this article will be made available by the authors, without undue reservation.

## Ethics Statement

The studies involving human participants were reviewed and approved by IRB 0801M23942 (University of Minnesota). The research consent requirement was waived because of the retrospective nature of the study.

## Author Contributions

YW conceived and designed the study. XT did radiomics analyses. CL and YW contributed to the data collection and analysis. DK made substantial contributions to the applications of machine learning techniques. DK and YW prepared the manuscript. All authors contributed to the article and approved the submitted version. 

## Conflict of Interest

The authors declare that the research was conducted in the absence of any commercial or financial relationships that could be construed as a potential conflict of interest.

## References

[1] Davis FG Dolecek TA McCarthy BJ Villano JL Toward determining the lifetime occurrence of metastatic brain tumors estimated from 2007 United States cancer incidence data. Neuro Oncol (2012) 14:1171–7. 10.1093/neuonc/nos152 PMC3424213 22898372

[2] Lu-Emerson C Eichler AF Brain metastases. Continuum (Minneap Minn) (2012) 18:295–311. 10.1212/01.CON.0000413659.12304.a6 22810128

[3] Kondziolka D Martin JJ Flickinger JC Friedland DM Brufsky AM Baar J Long-term survivors after gamma knife radiosurgery for brain metastases. Cancer (2005) 104:2784–91. 10.1002/cncr.21545 16288488

[4] Andrews DW Scott CB Sperduto PW Flanders AE Gaspar LE Schell MC Whole brain radiation therapy with or without stereotactic radiosurgery boost for patients with one to three brain metastases: phase III results of the RTOG 9508 randomised trial. Lancet (2004) 363:1665–72. 10.1016/S0140-6736(04)16250-8 15158627

[5] Shaw E Scott C Souhami L Dinapoli R Kline R Loeffler J Single dose radiosurgical treatment of recurrent previously irradiated primary brain tumors and brain metastases: final report of RTOG protocol 90-05. Int J Radiat Oncol Biol Phys (2000) 47:291–8. 10.1016/s0360-3016(99)00507-6 10802351

[6] Sperduto PW Berkey B Gaspar LE Mehta M Curran W A new prognostic index and comparison to three other indices for patients with brain metastases: an analysis of 1,960 patients in the RTOG database. Int J Radiat Oncol Biol Phys (2008) 70:510–4. 10.1016/j.ijrobp.2007.06.074 17931798

[7] Sperduto PW Yang TJ Beal K Pan H Brown PD Bangdiwala A Estimating Survival in Patients With Lung Cancer and Brain Metastases: An Update of the Graded Prognostic Assessment for Lung Cancer Using Molecular Markers (Lung-molGPA). JAMA Oncol (2017) 3:827–31. 10.1001/jamaoncol.2016.3834 PMC5824323 27892978

[8] Lambin P Rios-Velazquez E Leijenaar R Carvalho S van Stiphout RG Granton P Radiomics: extracting more information from medical images using advanced feature analysis. Eur J Cancer (2012) 48:441–6. 10.1016/j.ejca.2011.11.036 PMC4533986 22257792

[9] Aerts HJ Velazquez ER Leijenaar RT Parmar C Grossmann P Carvalho S Decoding tumour phenotype by noninvasive imaging using a quantitative radiomics approach. Nat Commun (2014) 5:4006. 10.1038/ncomms5006 24892406 PMC4059926

[10] Mouraviev A Detsky J Sahgal A Ruschin M Lee YK Karam I Use of Radiomics for the Prediction of Local Control of Brain Metastases After Stereotactic Radiosurgery. Neuro Oncol (2020) 22(6):797–805. 10.1093/neuonc/noaa007 pii: noaa007. 31956919 PMC7283017

[11] Kumar V Gu Y Basu S Berglund A Eschrich SA Schabath MB Radiomics: the process and the challenges. Magn Reson Imaging (2012) 30:1234–48. 10.1016/j.mri.2012.06.010 PMC3563280 22898692

[12] Ahmed A Gibbs P Pickles M Turnbull L Texture analysis in assessment and prediction of chemotherapy response in breast cancer. J Magnetic Resonance Imaging (2013) 38:89–101. 10.1002/jmri.23971 23238914

[13] Wu W Parmar C Grossmann P Quackenbush J Lambin P Bussink J Exploratory Study to Identify Radiomics Classifiers for Lung Cancer Histology. Front Oncol (2016) 6:71. 10.3389/fonc.2016.00071 27064691 PMC4811956

[14] Fried DV Tucker SL Zhou S Liao Z Mawlawi O Ibbott G Prognostic value and reproducibility of pretreatment CT texture features in stage III non-small cell lung cancer. Int J Radiat Oncol Biol Phys (2014) 90:834–42. 10.1016/j.ijrobp.2014.07.020 PMC4349397 25220716

[15] Itakura H Achrol AS Mitchell LA Loya JJ Liu T Westbroek EM Magnetic resonance image features identify glioblastoma phenotypic subtypes with distinct molecular pathway activities. Sci Transl Med (2015) 7:303ra138. 10.1126/scitranslmed.aaa7582 PMC4666025 26333934

[16] Kickingereder P Götz M Muschelli J Wick A Neuberger U Shinohara RT Large-scale Radiomic Profiling of Recurrent Glioblastoma Identifies an Imaging Predictor for Stratifying Anti-Angiogenic Treatment Response. Clin Cancer Res (2016) 22:5765–71. 10.1158/1078-0432.CCR-16-0702 PMC5503450 27803067

[17] Zhou M Scott J Chaudhury B Hall L Goldgof D Yeom KW Radiomics in Brain Tumor: Image Assessment, Quantitative Feature Descriptors, and Machine-Learning Approaches. AJNR Am J Neuroradiol (2018) 39:208–16. 10.3174/ajnr.A5391 PMC5812810 28982791

[18] Ryu YJ Choi SH Park SJ Yun TJ Kim JH Sohn CH Glioma: application of whole-tumor texture analysis of diffusion-weighted imaging for the evaluation of tumor heterogeneity. PloS One (2014) 9:e108335. 10.1371/journal.pone.0108335 25268588 PMC4182447

[19] Rizzo S Petrella F Buscarino V De Maria F Raimondi S Barberis M CT Radiogenomic Characterization of EGFR, K-RAS, and ALK Mutations in Non-Small Cell Lung Cancer. Eur Radiol (2016) 26:32–42. 10.1007/s00330-015-3814-0 25956936

[20] Huang CY Lee CC Yang HC Lin CJ Wu HM Chung WY Radiomics as prognostic factor in brain metastases treated with Gamma Knife radiosurgery. J Neurooncol (2020) 146(3):439–49. 10.1007/s11060-019-03343-4 32020474

[21] Goodman KA Sneed PK McDermott MW Shiau CY Lamborn KR Chang S Relationship between pattern of enhancement and local control of brain metastases after radiosurgery. Int J Radiat Oncol Biol Phys (2001) 50:139–46. 10.1016/s0360-3016(00)01584-4 11316557

[22] Lin HY Watanabe Y Cho LC Yuan J Hunt MA Sperduto PW Gamma knife stereotactic radiosurgery for renal cell carcinoma and melanoma brain metastases-comparison of dose response. J Radiosurg SBRT (2013) 2:193–207. 29296362 PMC5658811

[23] Eisenhauer EA Therasse P Bogaerts J Schwartz LH Sargent D Ford R New response evaluation criteria in solid tumours: revised RECIST guideline (version 1.1). Eur J Cancer (2009) 45:228–47. 10.1016/j.ejca.2008.10.026 19097774

[24] Zhang L Fried DV Fave XJ Hunter LA Yang J Court LE IBEX: an open infrastructure software platform to facilitate collaborative work in radiomics. Med Phys (2015) 42:1341–53. 10.1118/1.4908210 PMC5148126 25735289

[25] Nieder C Berberich W Schnabel K Tumor-related prognostic factors for remission of brain metastases after radiotherapy. Int J Radiat Oncol Biol Phys (1997) 39:25–30. 10.1016/s0360-3016(97)00154-5 9300736

[26] Zhang Z Yang J Ho A Jiang W Logan J Wang X A predictive model for distinguishing radiation necrosis from tumour progression after gamma knife radiosurgery based on radiomic features from MR images. Eur Radiol (2018) 28(6):2255–63. 10.1007/s00330-017-5154-8 PMC6036915 29178031

[27] Peng L Parekh V Huang P Lin DD Sheikh K Baker B Distinguishing True Progression From Radionecrosis After Stereotactic Radiation Therapy for Brain Metastases With Machine Learning and Radiomics. Int J Radiat Oncol Biol Phys (2018) 102(4):1236–43. 10.1016/j.ijrobp.2018.05.041 PMC6746307 30353872

[28] Zhang B Lian Z Zhong L Zhang X Dong Y Chen Q Machine-learning based MRI radiomics models for early detection of radiation-induced brain injury in nasopharyngeal carcinoma. BMC Cancer (2020) 20(1):502. 10.1186/s12885-020-06957-4 32487085 PMC7268644

[29] Kocher M Ruge MI Galldiks N Lohmann P Applications of radiomics and machine learning for radiotherapy of malignant brain tumors. Strahlenther Onkol (2020) 196(10):856–67. 10.1007/s00066-020-01626-8 PMC7498494 32394100

[30] Wu J Aguilera T Shultz D Gudur M Rubin DL Loo BW Jr Early-Stage Non-Small Cell Lung Cancer: Quantitative Imaging Characteristics of (18)F Fluorodeoxyglucose PET/CT Allow Prediction of Distant Metastasis. Radiology (2016) 281:270–8. 10.1148/radiol.2016151829 PMC5047129 27046074

